# Recurrence of COVID-19 after recovery: a case report from Italy

**DOI:** 10.1007/s15010-020-01444-1

**Published:** 2020-05-16

**Authors:** Daniela Loconsole, Francesca Passerini, Vincenzo Ostilio Palmieri, Francesca Centrone, Anna Sallustio, Stefania Pugliese, Lucia Donatella Grimaldi, Piero Portincasa, Maria Chironna

**Affiliations:** 1 Department of Biomedical Sciences and Human Oncology-Hygiene Section, University of Bari, “Aldo Moro”, Piazza G. Cesare 11, 70124 Bari, Italy; 2 Department of Biomedical Sciences and Human Oncology-Clinica Medica “A. Murri”, University of Bari, Piazza G. Cesare 11, 70124 Bari, Italy; 3 Azienda Ospedaliero-Universitaria Consorziale Policlinico di Bari, Piazza G. Cesare 11, 70124 Bari, Italy

Dear Editors,

Since the diffusion of SARS-CoV-2 infection outside China, Italy became one of the world’s worst-affected country. By May 3, 2020, recorded cases in Italy were 210,717, with 28,884 deaths and 81,654 recovered cases.

Here, we describe a case of reactivation of COVID-19 registered in Italy at the beginning of May 2020.

**Fig. 1:**
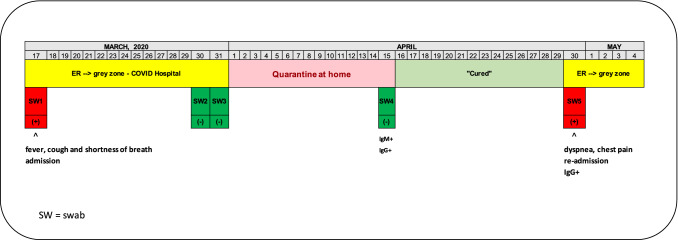
Timeline of SARS-CoV-2 infection

On March 17, a 48-year-old man visited the Emergency Department, Policlinico Hospital of Bari, Puglia region (Italy), with fever, cough and shortness of breath, hyporexia for 6 days (Fig. 1). Physical examination revealed normal vital signs but because of 90% oxygen saturation on ambient air, the patient was promptly treated with O_2_ 6 lt/min (Venturi Mask 31%). The patient did not report any underlying medical condition such as diabetes, hypertension, or cardiovascular disease. For the suspicion of COVID-19, he was immediately admitted to the “grey zone” of internal medicine, at the “Asclepios” COVID-Hospital, Policlinico. The chest X-ray showed a pneumonia (bilateral multiple thickenings with badly defined margins with consolidation aspects more evident on the right side). The real-time PCR on the nasopharyngeal swab collected on March 18 revealed the presence of SARS-CoV-2. The virus was detected by a real-time PCR assay targeting E-gene, RdRP-gene and N-gene, performed with the protocol previously reported by the WHO (https://www.who.int/docs/default-source/coronaviruse/uscdcrt-pcr-panel-for-detection-instructions.pdf?sfvrsn=3aa07934_2). Based on the criteria of Wang et al. (2020), the patient had a severe form of the disease due to the presence of fever, respiratory symptoms, radiological signs of pneumonia and PaO_2_/FiO_2_ < 300 mmHg [1]. He was treated with O_2_ at different volumes (up to 60% FiO_2_ VM), lopinavir/ritonavir (200/50 mg, 2 tablets × 2/day), hydroxychloroquine (400 mg b.i.d on the first day, and 200 mg b.i.d afterwards), enoxaparin 6000 IU b.i.d., methylprednisolone (starting dose 40 mg b.i.d, lately tapered). At the checkup after 6 days, the chest X-ray showed a slight improvement involvement.

After 14 days the patients became afebrile and his respiratory symptoms disappeared. The chest X-ray showed only blurred areas of parenchymal thickening. Our hospital required two consecutive negative SARS-CoV-2 molecular tests, plus normal body temperature, resolution of respiratory symptoms, with the improvement of lung imaging. The two nasopharyngeal swabs collected on March 30 and 31 were both negative for SARS-CoV-2 infection. The patient was therefore discharged and encouraged to maintain home quarantine for at least 14 days. The molecular test was also negative at his follow-up visit on April 15, suggesting that the patient was cured from COVID-19. In addition, two serological assays (VivaDiag™, VivaChek Laboratories, INC, USA and Anti SARS-CoV-2 ELISA IgG Test, Euroimmun, Lubeck, Germany) revealed the presence of IgM and IgG anti-SARS-CoV-2. However, on April 30, he developed new symptoms, i.e., dyspnea and chest pain. He visited again the Emergency Department where he was re-admitted to the same ward with a suspicion of a pulmonary embolism that was confirmed by CT scan. The imaging showed the presence of segmental and sub-segmental signs of arterial microembolism with some parcel area of ground glass. Because of his recent clinical history, a SARS-CoV-2 molecular test was performed and proved to be positive. Moreover, serological assay revealed the presence of only IgG anti-SARS-CoV-2. To date, the patient is well, on anticoagulant therapy and does not require O_2_ supplementation.

To the best of our knowledge, this is the first published report describing a reactivation of COVID-19 in an apparently cured patient in Italy.

The presence of the virus in infected patient seems to be fluctuant because of the possible occurrence of false-negative results at molecular test, because of viral load, the experience of the operator in collecting the sample and to the sampling site [2]. Nevertheless, the case we describe points to a real reactivation of the infection since the molecular test became positive again following three previous negative tests in one month. In a recent paper, Ye et al. reported a 9% proportion of reactivation in COVID-19 patients after discharge from hospital [3]. Risk factors of reactivation would probably include host status, virologic features and, for example, steroid-induced immunosuppression [3]. The possibility of a reactivation of COVID-19 poses a major public health concern since it could significantly contribute to the spread of the virus in the population. Domiciliary quarantine of 14 days applies to all COVID-19 patients after hospital discharge, but a clear definition of the infectiousness timing and duration of viral shedding is still lacking [4]. Pre-symptomatic and asymptomatic carriers may be infectious [5], but we should consider that also the convalescent may transmit the virus [2]. Further investigations should better define the most appropriate quarantine period, to avoid transmission [4]. This case had anti-SARS-CoV-2 IgG, indicating that the acute phase of the disease was exceeded. Preliminary evidences suggest that antibody responses occur in those who have been infected [6]. If these antibodies are protective and how long their protection will last, is yet to be established. According to the present report, we could speculate that in some cases the presence of IgG antibodies is not protective.

In conclusion, the ongoing public health emergency requires additional and urgent investigations on convalescent cases, to contain the pandemic. This policy should limit the further viral spread in the population, preventing an increase in number of cases and deaths.
